# Responses of Soil Microbial Communities and Functions in an Alpine Grassland of the Qinghai Lake Basin With Grazing Disturbance

**DOI:** 10.1002/ece3.71082

**Published:** 2025-03-23

**Authors:** Caicai Sun, Quanmin Dong, Haitao An, Yuzhen Liu, Weidong Lv, Wenting Liu, Haiming Ji, Xiaoxia Yang

**Affiliations:** ^1^ Academy of Animal Science and Veterinary Medicine Qinghai University Xining China; ^2^ Qinghai Provincial Key Laboratory of Adaptive Management on Alpine Grassland Xining China; ^3^ Key Laboratory of Alpine Grassland Ecosystem in the Three‐River‐Source Ministry of Education, Qinghai University Xining China

**Keywords:** alpine grassland, community function, grazing, soil microbial community structure

## Abstract

Soil ecosystems host diverse microbial communities, which are influenced by various environmental factors, soil properties, vegetation characteristics, and anthropogenic activities, such as livestock grazing. Grazing serves as a critical management practice in the alpine grasslands of the Qinghai‐Tibet Plateau, affecting soil microbial communities and their functions through processes such as forage consumption, trampling, and the deposition of feces and urine. In this study, we utilized the scientific and technological platform “Alpine Grassland‐Livestock Adaptive Management Technology Platform” in Qinghai Province to examine the effects of grazing intensity on soil microbial communities and functions. Experimental treatments included different grazing intensities (light grazing, moderate grazing, and heavy grazing), along with a no‐grazing control. Metagenomic sequencing technology was employed to investigate the impact of these grazing intensities on the microbial community composition and functional attributes in alpine grasslands. The results indicated that: (1) Actinobacteria, Proteobacteria, and Chloroflexi were the dominant bacterial communities in the soil, while Ascomycota, Mucoromycota, and Basidiomycota represented the primary fungal communities. (2) Grazing had a greater impact on soil fungal communities than on bacterial communities, altering the Shannon diversity index and Simpson index of soil fungal communities. (3) Soil pH and soil moisture were important factors influencing changes in soil microbial communities. (4) Functional analysis focusing on the “nitrogen metabolism” pathway indicated that under light grazing conditions, the relative abundance of multiple functional genes, particularly those involved in denitrification, decreased.

## Graphical abstract

The study investigated the response of soil microorganisms in alpine grassland to different grazing intensities in the Qinghai‐Tibet Plateau, elucidating the changes in composition and diversity of soil microorganisms among different treatments. Additionally, the nitrogen metabolism pathway was specifically examined.

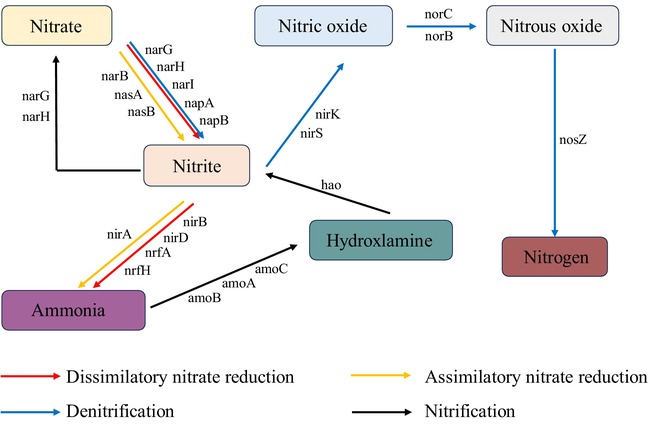

## 1 Introduction

The Qinghai‐Tibet Plateau not only possesses a unique geographical location but also abundant biological resources. Alpine grassland, accounting for approximately 60% of the Plateau's total area, is one of the most unique grassland ecosystems globally and plays a crucial role in maintaining biodiversity and regional economic development on the Qinghai‐Tibet Plateau (H. K. Wang et al. 2022). Grazing is a primary method of utilizing alpine grasslands on the Qinghai‐Tibet Plateau (Yang et al. 2013). However, the alpine grassland on the Qinghai‐Tibet Plateau has experienced degradation to varying degrees as a result of climate change, grazing, and other factors (Zhang et al. 2016). Long‐term overgrazing can cause irreversible damage to alpine grasslands, but solely restricting grazing would affect the economic development of the Qinghai‐Tibet Plateau region. Studies have shown that the degree of grassland degradation and the decline in soil nutrient quality were influenced by different grazing intensities (Ren 2012). Additionally, Hong et al. (2020) Study showed that forbidding grazing reduced soil acidity, especially in topsoil. Different grazing intensities altered the soil carbon‐nitrogen ratio, with light grazing increased the ratio, and heavy grazing decreased it (He et al. 2019). In fact, the impact of grazing on ecosystems was very complex, with different climatic conditions and grazing times both influencing the ultimate outcome (Wang et al. 2018; Eldridge et al. 2016). So far, numerous scholars have conducted extensive research on the impact of grazing on soil physical and chemical properties (Török et al. 2018), but relatively few studies have investigated the effects of grazing on soil microbial community. Evaluating the optimal grazing intensity that promotes soil ecosystem health and sustainable development is of critical importance for maintaining ecological stability and ensuring sustainable development in the Qinghai‐Tibet Plateau region.

Soil microorganisms are an indispensable component of the grassland ecosystem, playing an important regulatory role in energy flow, material input, and output processes within the system (Moll et al. 2017). As an important biological medium at the plant–soil interface, soil microorganisms influence the cycling process of soil nutrients through decomposition and directly interact with plant roots, thereby influencing plant growth and community succession (Li et al. 2016). Grazing livestock's hoof impacts compact the soil and alter conditions such as water potential and ventilation. Meanwhile, their feces and urine excretion enhances soil nutrient availability and increase easily decomposable carbon content, affecting the diversity and functionality of soil microbial communities (Eldridge and Delgado‐Baquerizo 2017). Moreover, the foraging activities of grazing livestock may cause plant biomass to be allocated towards belowground and increase root exudation, impacting soil microbial community structure (Mueller et al. 2017). As grazing intensity increased, grassland degradation intensified, soil quality declined, and the living environment of soil microorganisms was threatened, often leading to a decrease in their numbers (Kooch et al. 2020). Furthermore, some microorganisms were unable to adapt to the new living environment and their numbers decreased or even disappeared completely, while some new microorganisms were introduced into the soil through livestock excretion, enhancing soil microorganism diversity (Qu et al. 2016). Studies showed that moderate grazing enhances soil bacterial community diversity, while light grazing promotes an increase in the diversity of soil arbuscular mycorrhizal fungi community (Ba et al. 2012). A global‐scale study on grazing and soil microbial communities found that light grazing promoted soil microbial richness, with a more pronounced increase in fungal community richness that in bacterial communities, while heavy grazing significantly reduced microbial richness (Wang and Tang 2019). Yang's study indicated that heavy grazing favored fast‐growing plant species, which promoted bacterial community growth, while light grazing favored slow‐growing plant species, which were beneficial to fungal community growth (F. Yang et al. 2019). Grazing altered the soil microbial composition, shifting from a fungal‐dominated to a bacterial‐dominated community and from slow‐growing to fast‐growing microorganisms, leading to a transition from a fungal‐dominated food web, based on recalcitrant organic carbon, to a bacterial‐dominated food web, reliant on unstable organic carbon (Xun et al. 2018). In fact, soil microorganisms are highly diverse; however, only a small fraction of them had been cultivated. Although the quantity and types of soil microorganisms in alpine grassland ecosystems provide some indication of the degree and type of grazing disturbance, their high diversity and largely unknown nature add complexity to related research. Furthermore, human interference has led to ongoing debate regarding the responses of soil microorganisms to grazing (Bardgett et al. 1997; Moussa et al. 2007).

To enhance understanding of soil microorganisms response to grazing intensity, this study conducted field experiments to explore: (1) whether different grazing intensities have distinct impacts on soil microbial communities; (2) which biological or non‐biological factors are influenced by varying grazing intensities, subsequently affecting soil bacterial and fungal communities; and (3) whether soil nitrogen metabolic processes change with different grazing intensities, given that the primary components of grazing livestock's feces and urine are water and nitrogen. This research provides a new perspective for the scientific utilization and management of alpine grasslands.

## 2 Materials and Methods

### 2.1 Study Site

This research is based on the scientific and technological basic condition platform “Alpine Grassland‐Livestock Adaptive Management Technology Platform”. The experimental area locates in Xihai Town, Haiyan County, Haibei Tibetan Autonomous Prefecture, Qinghai Province, China (36°44′–37°39′ N, 100°23′–101°20′ E, altitude 3150 m). This region is characterized by a plateau continental climate with an average annual temperature of about 1.5°C and an average annual rainfall of about 400 mm. Precipitation is concentrated mainly from June to September, and the plant growth period lasts from May to September. The soil type is sandy loam, and the grassland type is alpine steppe meadow. The plant community dominates by *Stipa purpurea*, *Kobresia humilis*, *Carex aridula*, and *Potentilla acaulis* (X. X. Yang et al. 2019).

### 2.2 Experimental Design

The experimental site was established in 2018, using alpine grasslands with uniform baseline conditions. A random block design was implemented, with four grazing treatments: no grazing (CK, plot area: 0.05 hm^2^, stocking rate: 0 Tibetan sheep·hm^−2^, grassland utilization rate: 0%), light grazing (LG, plot area: 0.22 hm^2^, stocking rate: 3.03 Tibetan sheep·hm^−2^, grassland utilization rate: 30%–35%), moderate grazing (MG, plot area: 0.17 hm^2^, stocking rate: 3.86 Tibetan sheep·hm^−2^, grassland utilization rate: 50%–55%), and heavy grazing (HG, plot area: 0.13 hm^2^, stocking rate: 5.13 Tibetan sheep·hm^−2^, grassland utilization rate: 65%–70%). Each treatment is replicated three times, resulting in a total of 12 experimental plots (Table 1). Since the establishment of the site, grazing is carried out from June to October every year. There are 2 Tibetan sheep in each grazing plot, and the grazing intensity is adjusted by varying the area of each grazing plot. To ensure consistency in grazing, the livestock used are male Tibetan sheep (one year old, weighing 30 ± 2 kg). During the grazing period, no supplementary feeding is provided to the Tibetan sheep in any of the grazing plots, and only the drinking water required by the Tibetan sheep is supplemented daily. The livestock were dewormed before grazing, and no supplementary feeding was provided during the entire grazing period (Zhou et al. 2023).

**TABLE 1:** Experimental design of grazing intensity.

Treatments	Number of Tibetan sheep·head^−1^	Area of plot·hm^−2^	Stocking rate (Tibetan sheep·hm^−2^)	Grassland utilization rate
No grazing, CK	0	0.05	0	0
Light grazing, LG	2	0.22	3.03	30%–35%
Moderate grazing, MG	2	0.17	3.86	50%–55%
Heavy grazing, HG	2	0.13	5.13	65%–70%

### 2.3 Sample Collection and Measurement

#### 2.3.1 Sample Collection

The vegetation survey and soil sample collection were conducted simultaneously in late July 2022. Within each grazing plot, three 0.5 × 0.5 m quadrats were randomly selected for investigation. The vegetation within each quadrat was cut at ground level, killed at 105°C for one hour, and then dried at 70°C to a constant weight to obtain the aboveground biomass (AGB). Afterward, a root auger with a diameter of 7 cm was used to collect samples from the 0–10 cm soil layer within each quadrat. The collected samples were thoroughly rinsed to obtain plant root samples, which were then dried to a constant weight to determine the belowground biomass (BGB).

Soil samples were collected from 20 randomly selected points in each grazing plot, with a sampling depth of 10 cm. The collected soil samples were mixed evenly, and impurities such as stones and roots were eliminated. One part of the soil was used for the determination of soil physical and chemical properties, while another part of the soil samples was immediately subjected to metagenomic sequencing.

#### 2.3.2 Sample Measurement

Determination of soil physical and chemical properties: Soil total potassium (TK) and available potassium (AK) were determined by the flame photometric method; Soil total phosphorus (TP) was determined by the molybdenum antimony anti‐colorimetry method; Soil total carbon (TC) and total nitrogen (TN) were determined by the elemental analyzer method; Soil available phosphorus (AP) was determined by the 0.5 mol·L^−1^ NaHCO_3_ method; Soil ammonium nitrogen (${\mathrm{NH}}_{4}^{+}‐N$) and nitrate nitrogen (${\mathrm{NO}}_{3}^{-}‐N$) were determined by the cutting‐edge analyzer; Soil pH (pH) was determined by potentiometry; Soil moisture (SM) was determined by the drying method; and Soil bulk density (BD) was determined by using a soil ring knife.

Based on the manufacturer's instructions, the genomic DNA of the samples was extracted using the HiPure kit (Guangzhou, China), and the quality of the DNA was tested. Genomic DNA that met the quality standards was fragmented by sonic degradation to obtain uniform 350 bp fragments. Subsequently, end repair, A‐tailing, and addition of Illumina sequencing adapters were performed using the NEBNext Ultra DNA Library Prep Kit (NEB, USA). Finally, the PCR products formed after amplification and enrichment of 300–400 bp DNA fragments were purified using the AMPure XP system (Beckman Coulter, Brea, CA, USA). The sequencing library was inspected using an Agilent 2100 Bioanalyzer (Agilent, Santa Clara, CA), and the library was quantified by real‐time PCR. Metagenomic sequencing was performed using the Illumina Novaseq 6000 sequencer, with the entire process employing a PE 150 strategy.

To obtain high‐quality clean reads, the raw data from the Illumina platform were filtered using FASTP (version 0.18.0). First, reads containing unknown nucleotides (N) ≥ 10% were filtered out. Second, reads with phred quality scores ≤ 20 and ≥ 50% were filtered out. Third, reads containing adapters were filtered out. The final clean reads were used for subsequent assembly analysis. The clean reads of the samples were assembled using MEGAHIT (version 1.1.2) to obtain continuous long sequences, contigs. Gene prediction was performed on contigs (> 500 bp) using MetaGeneMark (version 3.38). Sequences with a similarity ≥ 95% and read coverage > 90% were merged into a cluster using CD‐HIT (version 4.6), and the longest sequence in each cluster was taken as the representative sequence (Unigene). Reads were realigned with Unigene using Bowtie (version 2.2.5) and their numbers were counted. After reading and filtering, a non‐redundant gene set was obtained. To fully explore the functional information of the community, annotate gene sequences using multiple databases, such as the Kyoto Encyclopedia of Genes and Genomes (KEGG).

### 2.4 Data Analysis

Using R version 4.0.2, we conducted a one‐way ANOVA to test the effects of different grazing intensities on soil physicochemical characteristics, followed by multiple comparisons using the Duncan method (*p* < 0.05). Additionally, the top 10 soil bacterial and fungal phyla were selected to visually demonstrate microbial changes. The alpha diversity of soil microbial communities was assessed using the Simpson and Shannon‐Wiener indices. Species differences between treatments were analyzed using Linear Discriminant Analysis Effect Size (LefSe) based on the platform at https://www.omicsmart.com, which was employed to identify the main microbial groups driving changes in soil microbial communities under different grazing intensities (Segata et al. 2011). Bray‐Curtis distances were calculated using the R vegan package and subsequently visualized using the R ggplot2 package to analyze the beta diversity of soil microorganisms. Functional annotation of soil microorganisms was performed based on the KEGG database, and Circos plots were generated using the R circlize package to illustrate functional differences in soil microorganisms among different treatments.

## 3 Results

### 3.1 Effects of Gazing Intensity on Soil Properties and Plant Properties

Besides total soil carbon (TC), other soil and plant properties were significantly different among the grazing intensities (*p* < 0.05). Specifically, grazing significantly decreased soil ammonium nitrogen (${\mathrm{NH}}_{4}^{+}‐N$), soil moisture (SM), microbial biomass carbon (MBC), and aboveground biomass (AGB) (*p* < 0.05) and significantly increased soil bulk density (BD) and soil pH (pH) (*p* < 0.05) (Table 2).

**TABLE 2:** Effects of grazing intensity on biotic and abiotic properties.

Variables	Treatments			
	CK	LG	MG	HG
Soil total nitrogen (TN, g·kg^−1^)	3.98 ± 0.15^a^	3.64 ± 0.05^ab^	3.57 ± 0.05^ab^	3.25 ± 0.38^b^
Soil total carbon (TC, g·kg^−1^)	49.82 ± 4.12^a^	43.28 ± 0.61^a^	43.54 ± 0.77^a^	39.43 ± 4.62^a^
Soil nitrate nitrogen (${\mathrm{NO}}_{3}^{-}‐N$, mg·kg^−1^)	0.53 ± 0.04^ab^	0.55 ± 0.02^ab^	0.60 ± 0.04^a^	0.43 ± 0.04^b^
Soil ammonium nitrogen (${\mathrm{NH}}_{4}^{+}‐N$, mg·kg^−1^)	10.88 ± 0.60^a^	5.27 ± 0.58^b^	6.15 ± 1.66^b^	5.34 ± 0.73^b^
Soil pH	8.10 ± 0.01^d^	8.32 ± 0.02^b^	8.41 ± 0.40^a^	8.24 ± 0.01^c^
Soil moisture content (SM, %)	19.00 ± 0.29^a^	13.41 ± 0.18^c^	14.79 ± 0.25^b^	15.35 ± 0.31^b^
Soil bulk density (BD, g·cm^−3^)	0.99 ± 0.02^b^	1.07 ± 0.01^a^	1.08 ± 0.01^a^	1.08 ± 0.03^a^
Microbial biomass carbon (MBC, mg·kg^−1^)	773.63 ± 7.07^a^	610.91 ± 11.99^b^	484.44 ± 36.72^c^	454.37 ± 19.61^c^
Microbial biomass nitrogen (MBN, mg·kg^−1^)	176.23 ± 5.01^a^	139.70 ± 22.03^ab^	110.37 ± 8.84^b^	99.51 ± 5.16^b^
Aboveground biomass (AGB, g·m^−2^)	170.71 ± 10.70^a^	140.56 ± 8.38^b^	106.99 ± 4.83^c^	72.43 ± 5.45^d^
Belowground biomass (BGB, g·m^−2^)	589.31 ± 182.81^a^	376.19 ± 68.98^ab^	330.12 ± 26.18^ab^	233.65 ± 20.59^b^

*Note:* Significant effects are indicated by different superscript indices. Significance level: *p* < 0.05.

Abbreviations: CK, no grazing; HG, heavy grazing; LG, light grazing; MG, moderate grazing.

### 3.2 Effects of Grazing Intensity on Bacterial and Fungal Community Compositions and Diversity

**FIGURE 1:**
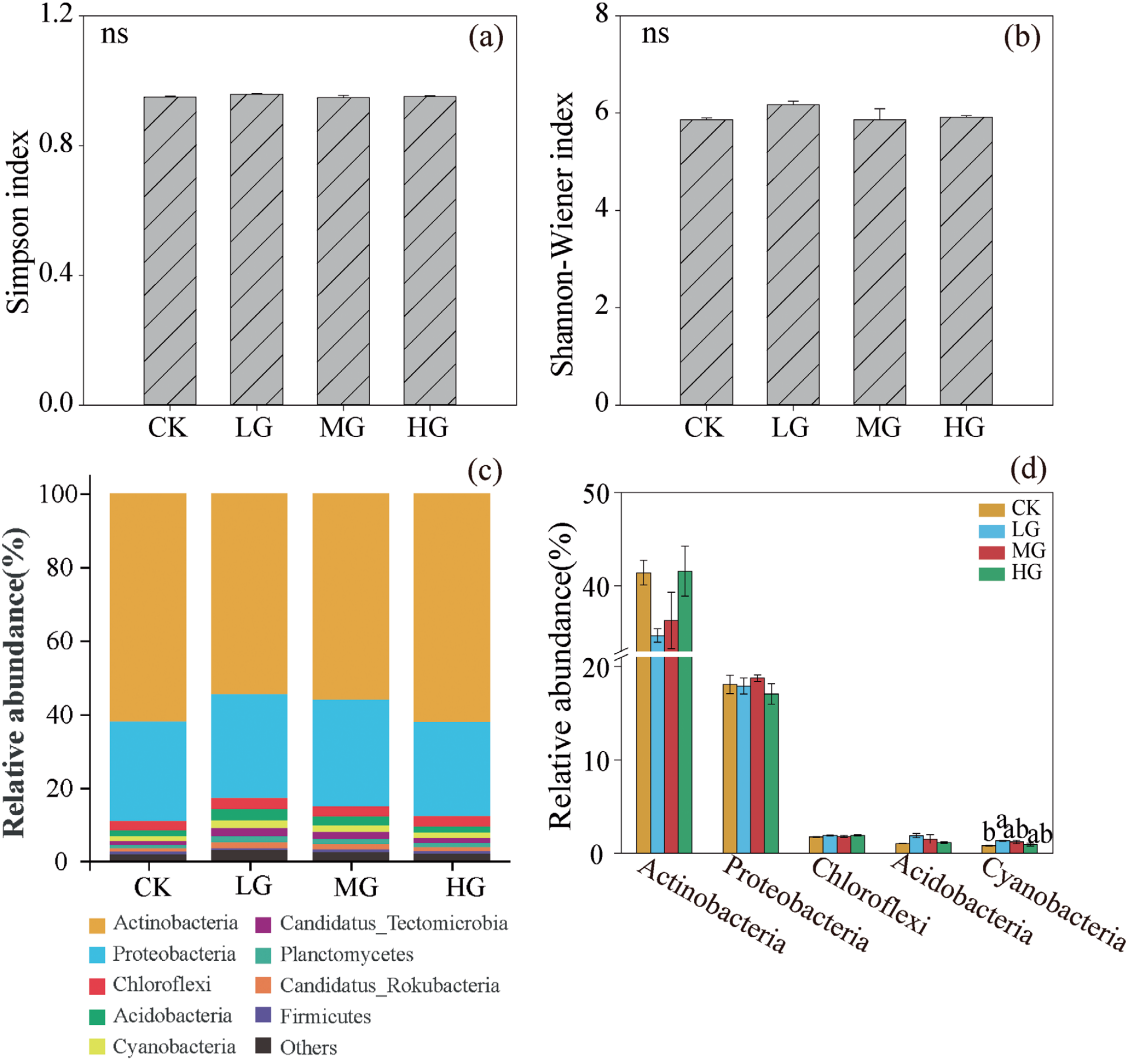
Comparison of soil bacterial communities. Species richness (a), Shannon‐Wiener index (b), taxonomic composition at the phylum level (c) and dominant bacterial phyla (d) of soil bacterial communities under different grazing intensities. Significance level: *p* < 0.05. CK, no grazing; HG, heavy grazing; LG, light grazing; MG, moderate grazing. The same below.

There were no significant differences in the Simpson and Shannon‐Wiener indices of soil bacteria under different grazing intensities (Figure 1a,b). The dominant phyla of soil bacteria belonged to Actinobacteria (34.67%–41.56%), Proteobacteria (17.07%–18.76%), and Chloroflexi (1.74%–1.92%) (Figure 1c). Among the bacteria with higher relative abundance, only Cyanobacteria showed differences between various grazing intensity treatments, with the relative abundance in the CK treatment being significantly lower than that in the LG treatment (*p* < 0.05) (Figure 1d). NMDS indicated that soil bacterial communities differed between different grazing intensity treatments (stress = 0.013) (Figure 3a).

**FIGURE 2:**
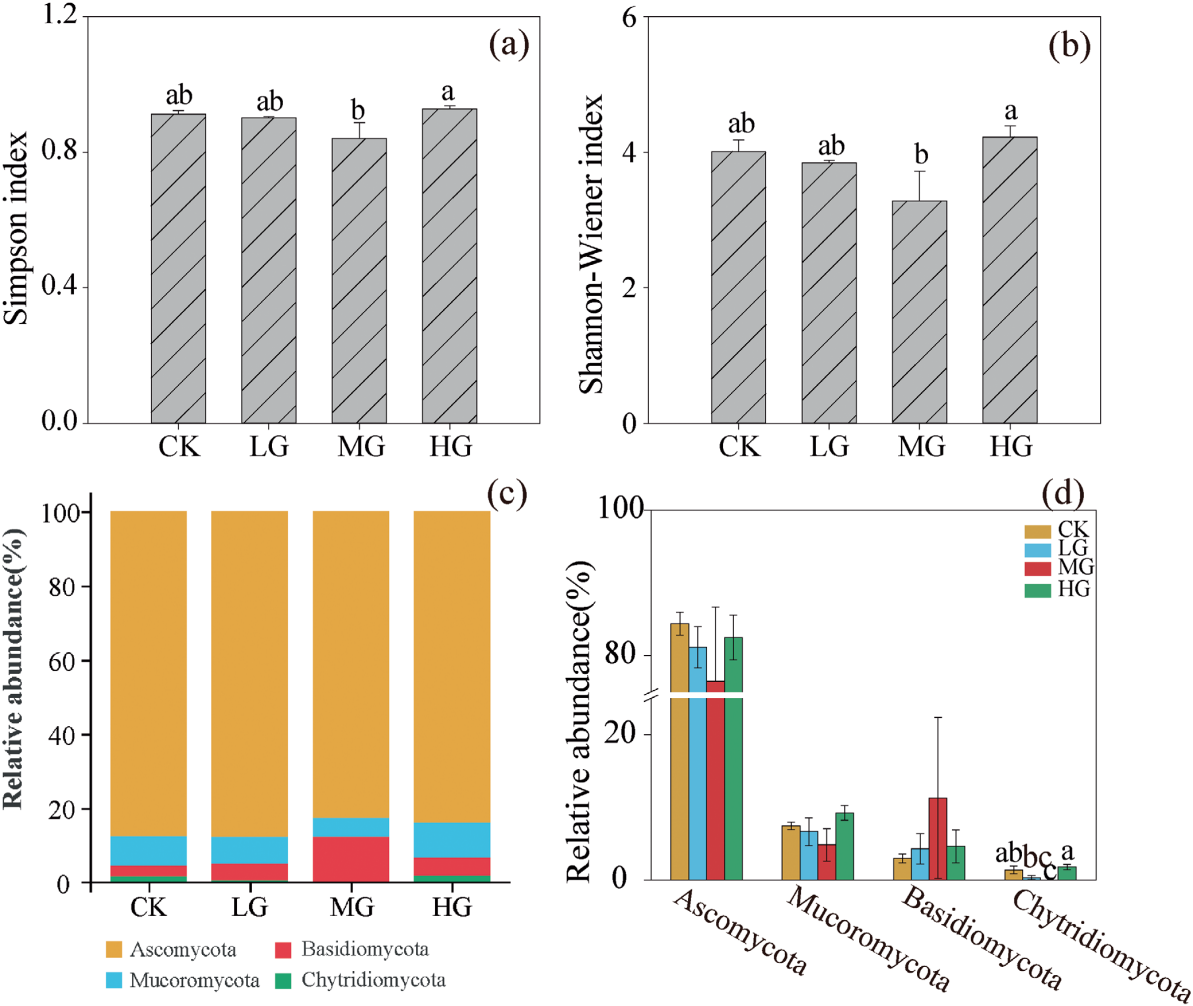
Comparison of soil fungal communities. Species richness (a), Shannon‐Wiener index (b), taxonomic composition at the phylum level (c) and dominant fungal phyla (d) of soil fungal communities under different grazing intensities.

**FIGURE 3:**
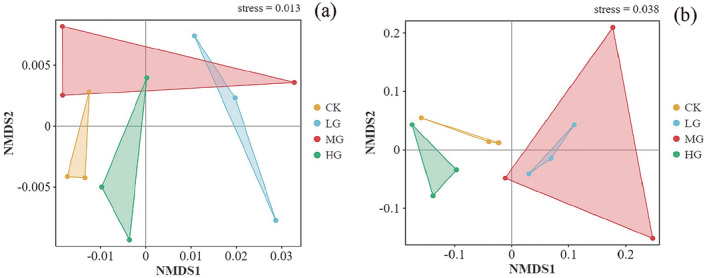
Nonmetric multidimensional scaling (NMDS) plot depicting the Bray‐Curtis distances of the bacterial community (a) and the fungal community (b).

Grazing had an impact on the Simpson and Shannon‐Wiener indices of soil fungi. The MG treatment decreased the Simpson and Shannon‐Wiener indices of soil fungi (*p* < 0.05) (Figure 2a,b). The dominant phyla of soil fungi belonged to Ascomycota (76.47%–84.37%), Mucoromycota (4.86%–9.25%), Basidiomycota (2.98%–11.30%), and Chytridiomycota (0.00%–1.82%) (Figure 2c). Analysis of fungi with higher relative abundance revealed that different grazing intensities had a significant impact on Chytridiomycota, with the MG treatment being significantly lower than the CK and HG treatments (*p* < 0.05) (Figure 2d). NMDS indicated that soil fungal communities differed between different grazing intensity treatments (stress = 0.038) (Figure 3b).

**FIGURE 4:**
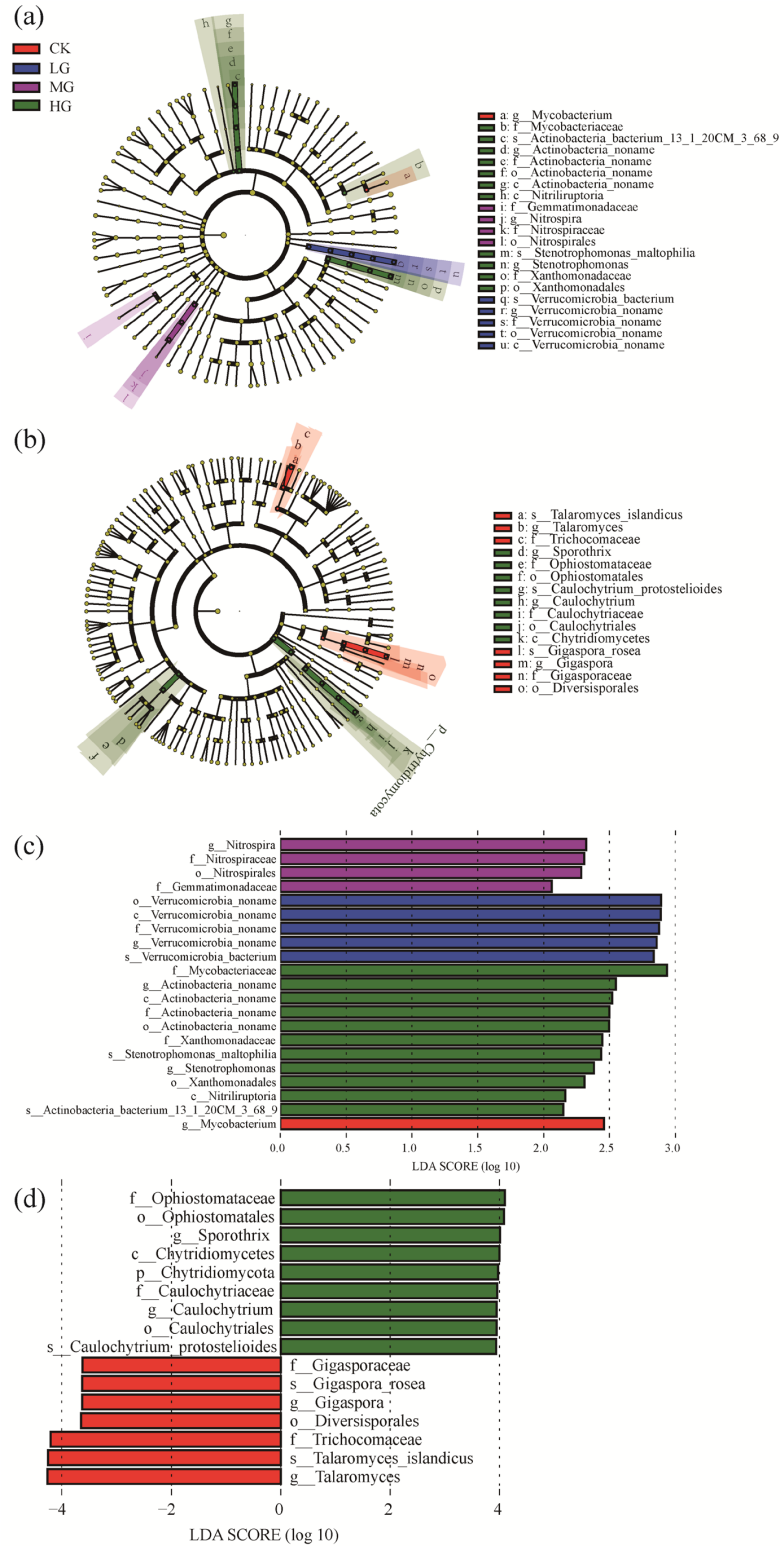
Cladogram indicates the phylogenetic distribution of microbial lineages under different grazing intensities, and each circle's diameter is proportional to the given taxon's relative abundance. Phylogenetic distribution of bacterial lineages under different grazing intensities (a); phylogenetic distribution of fungal lineages under different grazing intensities (b); indicator bacteria with linear discriminant analysis (LDA) scores of 2 or greater in bacterial communities under different grazing intensities (c); and indicator fungi with LDA scores of 2 or greater in fungal communities under different grazing intensities (d).

To identify the significantly different soil bacterial and fungal taxa under various grazing intensities, Linear Discriminant Analysis (LDA) Effect Size (LEfSe) analysis was conducted (Figure 4). The results revealed 21 significantly different bacterial species and 16 significantly different fungal species. In the CK treatment, *Mycobacterium* was the most abundant soil bacterial group, while *Gigasporaceae*, *Talaromuces*, and *Diversisporales* were the most abundant soil fungal groups. In the LG treatment, *Verrucomicrobia* was the most abundant soil bacterial group. In the MG treatment, *Nitrospirals* and *Gemmatimonadaceae* were the most abundant soil bacterial groups. In the HG treatment, *Mycobacteriaceae*, Actinobacteria, *Xanthomonadales*, and *Nitriliruptoria* were the most abundant soil fungal groups.

### 3.3 Correlations Between Biotic and Abiotic Properties and Soil Microbial Community

**FIGURE 5:**
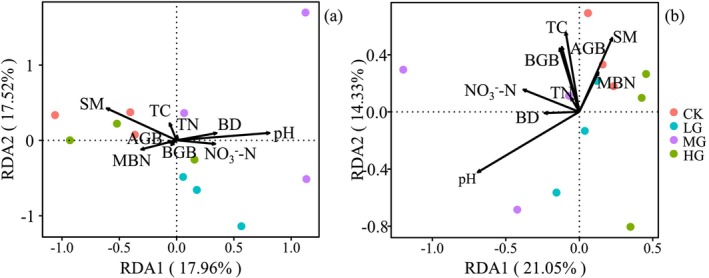
Canonical correspondence analysis using pooled data of bacterial (a) and fungal (b) communities and abiotic and biotic variables (arrows). AGB, aboveground biomass; BD, soil bulk density; BGB, belowground biomass; MBN, microbial biomass nitrogen; ${\mathrm{NO}}_{3}^{-}‐N$, soil nitrate nitrogen; pH, soil pH; SM, soil moisture content; TC, soil total carbon; TN, soil total nitrogen.

First, indicators with high collinearity were screened through correlation analysis (Table 3), followed by RDA analysis. The results showed that the eigenvalues of the two axes in the soil bacterial community were 35.48% (the eigenvalue of the first axis was 17.96%, and the eigenvalue of the second axis was 17.52%). pH and SM were significantly positively correlated with the composition of the soil bacterial community (Figure 5a and Table 4). The eigenvalues of the two axes in the soil fungal community were 35.38% (the eigenvalue of the first axis was 21.05%, and the eigenvalue of the second axis was 14.33%). pH was significantly positively correlated with the composition of the soil fungal community (Figure 5b and Table 4).

**TABLE 3:** Correlation analysis between biotic and abiotic properties.

	TN	TC	${\mathrm{NO}}_{3}^{-}‐N$	${\mathrm{NH}}_{4}^{+}‐N$	pH	SM	BD	BGB	AGB	MBC	MBN
TN	1										
TC	0.41	1									
${\mathrm{NO}}_{3}^{-}‐N$	−0.00	0.43	1								
${\mathrm{NH}}_{4}^{+}‐N$	0.59*	0.34	0.11	1							
pH	−0.29	−0.34	0.32	−0.68*	1						
SM	0.38	0.37	−0.18	0.83**	−0.79**	1					
BD	−0.37	−0.23	0.01	−0.76**	0.63*	−0.71**	1				
BGB	0.64*	0.62*	0.28	0.54	−0.44	0.44	−0.54	1			
AGB	0.31	0.36	0.12	0.37	−0.49	0.49	−0.35	0.75**	1		
MBC	0.53	0.58*	0.22	0.70*	−0.69*	0.64*	−0.73**	0.85**	0.62*	1	
MBN	0.50	0.61*	0.29	0.61*	−0.53	0.55	−0.64*	0.72**	0.50	0.91**	1

Abbreviations: AGB, aboveground biomass; BD, soil bulk density; BGB, belowground biomass; MBC, microbial biomass carbon; MBN, microbial biomass nitrogen; ${\mathrm{NH}}_{4}^{+}‐N$, soil ammonium nitrogen; ${\mathrm{NO}}_{3}^{-}‐N$, soil nitrate nitrogen; pH, soil pH; SM, soil moisture content; TC, soil total carbon; TN, soil total nitrogen.* represents *p* < 0.05, ** represents *p* < 0.01.

**TABLE 4:** Relationship between soil microbial community and biotic and abiotic variables.

Variables	Bacteria		Fungi	
	*r* ^2^	*p*	*r* ^2^	*p*
Soil total nitrogen (TN, g·kg^−1^)	0.00	0.98	0.01	0.90
Soil total carbon (TC, g·kg^−1^)	0.05	0.76	0.31	0.20
Soil nitrate nitrogen (${\mathrm{NO}}_{3}^{-}‐N$, mg·kg^−1^)	0.11	0.57	0.17	0.45
Soil pH	**0.66**	**0.00**	**0.62**	**0.01**
Soil moisture content (SM, %)	**0.53**	**0.03**	0.30	0.20
Soil bulk density (BD, g·cm^−3^)	0.13	0.50	0.05	0.78
Aboveground biomass (AGB, g·m^−2^)	0.01	0.97	0.21	0.35
Belowground biomass (BGB, g·m^−2^)	0.01	0.96	0.21	0.32
Microbial biomass nitrogen (MBN, mg·kg^−1^)	0.11	0.62	0.09	0.67

*Note:* Bold values represent significant relationships. Significance level: *p* < 0.1.

### 3.4 Functions of Soil Microbial Community

**FIGURE 6:**
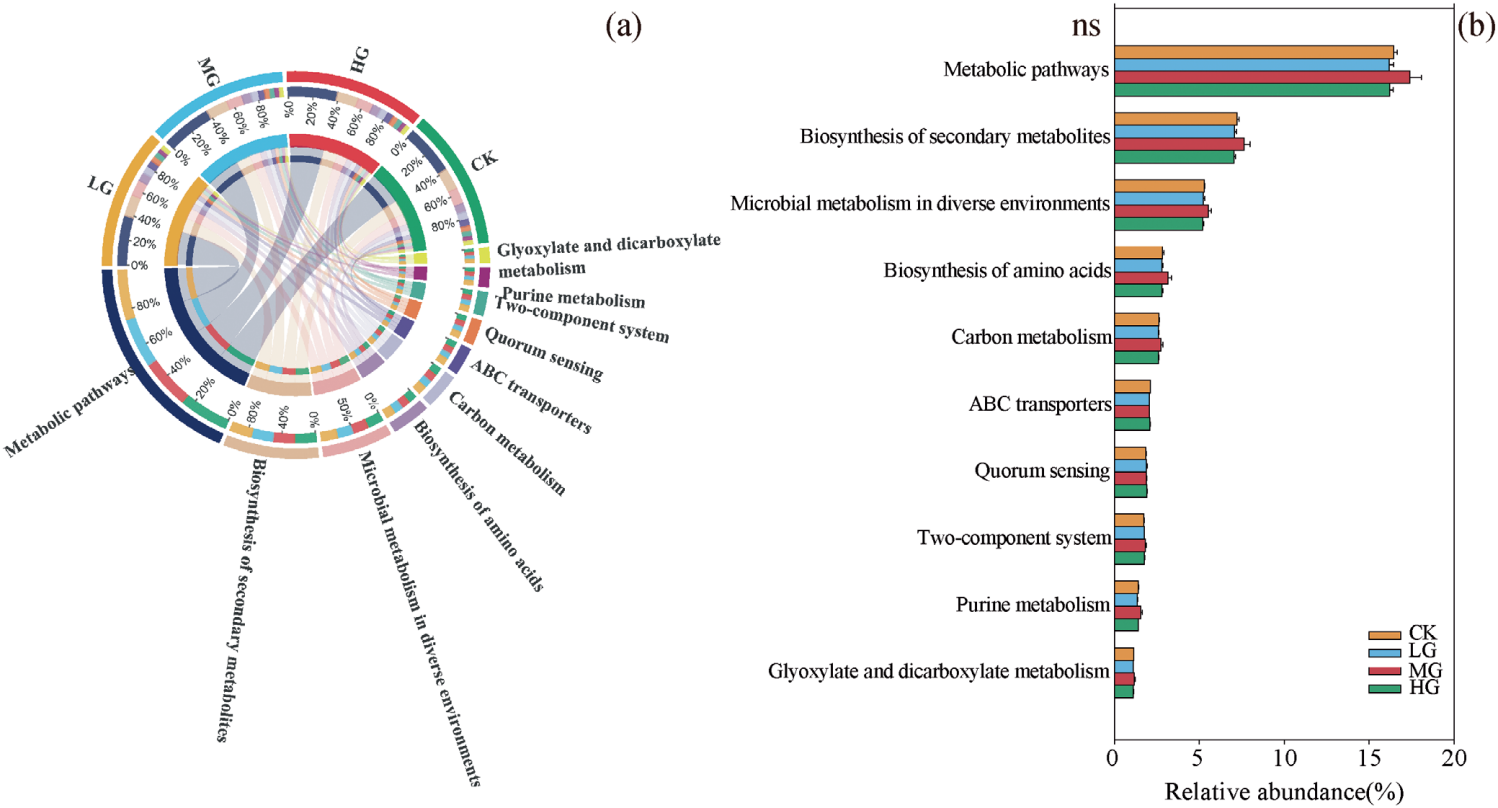
(a) Composition of main KEGG pathways of soil microorganisms under different grazing intensity treatments; (b) Comparison of KEGG pathway differences in soil samples between different grazing intensity treatments.

Through annotation analysis of microbial functions under different grazing intensities using the KEGG database, a total of 368 KEGG pathways were annotated. By analyzing the functional pathways with higher relative abundance (Figure 6a), the most abundant pathways were Metabolic pathways, Biosynthesis of secondary metabolites, and Microbial metabolism in diverse environments. There were no significant differences in the relative abundance of the higher KEGG pathways among different grazing intensities (Figure 6b). Additionally, KEGG functional annotation was performed on the genes of soil samples under different grazing intensities, further identifying genes related to soil nitrogen metabolism (Table S1). The research results showed that under different grazing intensities, various enzymes encoded by corresponding functional genes participated in the soil nitrogen metabolism process, and the relative abundance of related genes also changed among different grazing intensities (Table S2). In the nitrogen metabolism cycle pathway (Figure S1), the abundances of *NarH*, *NarI*, and *NrfA* genes were significantly lower in the LG treatment than in other treatments in the Dissimilatory nitrate reduction pathway; the functional genes *NarB* and *NasB* were significantly lower in the LG treatment than in other treatments in the Assimilatory nitrate reduction pathway, while the functional genes *NasA* and *NirA* were not significant across all grazing intensities; the functional genes *NarH*, *NarI*, *NorC*, and *NosZ* were the lowest in the LG treatment in the Denitrification pathway, and the NirS functional gene was the highest in the HG treatment and lowest in the LG treatment. The relative abundance of Nitrification genes, such as *narH*, *amoC*, and *amoA*, was lowest in the LG treatment.

## 4 Discussion

### 4.1 Effects of Different Grazing Intensities on Soil Microbial Communities

Soil microorganisms play a central role in processes such as soil nutrient cycling and organic matter decomposition and are essential for maintaining the health of grassland ecosystems (Wei et al. 2017). This study, utilizing metagenomic sequencing technology, identified differences in the composition of soil bacterial and fungal communities under different grazing intensities (Figures 1 and 2). The dominant phyla of soil bacteria were mainly composed of Actinobacteria and Proteobacteria, which were consistent with the results of previous studies (Gong et al. 2019; Qin et al. 2021). Actinobacteria was a gram‐positive bacterium, and most of their taxa were symbiotic. They usually dominated in nutrient‐rich environments, promoting the decomposition of organic matter and being closely related to the nitrogen cycle in nature (Górska et al. 2022). Proteobacteria was a major group of soil bacteria, including various nitrogen‐fixing bacteria, and were the dominant group of microorganisms in the plant rhizosphere. Both were beneficial bacterial phyla in the soil, facilitating plant nitrogen absorption and utilization (Jiang et al. 2016). The relative abundances of these dominant bacterial phyla varied somewhat but not significantly among different grazing intensities. A possible explanation is that litter and plant root exudates varied under different grazing treatments, with different plants producing distinct litter and root exudates, which subsequently enhanced the competitive ability among bacterial communities and resulted in differences in the relative abundances of soil bacteria. Soil fungi at the phylum level mainly included Ascomycota, Mucoromycota, and Basidiomycota, which were important groups of soil fungi and participated in the soil carbon cycle by degrading organic matter. Additionally, some groups within the Basidiomycota could form symbiotic relationships with algae and mosses and were also important decomposers of plant lignin (Kabuyah et al. 2012). The results of this study indicated that grazing had no significant effect on the relative abundances of dominant fungal groups within the Ascomycota, Mucoromycota, and Basidiomycota. This also suggested that despite different grazing intensities, the dominant groups of soil fungi exhibited a certain level of similarity. This, in turn, reflected that the dominant groups of soil fungi had good adaptability to different habitats and possessed strong anti‐interference ability against grazing.

Grazing was a potential factor affecting soil microbial diversity in grassland ecosystems. In this study, the Simpson index and Shannon‐Wiener index were used to reflect the overall diversity of soil microbial communities. It was found that the diversity indices of soil bacteria were not significantly different among different grazing intensities, while the MG treatment decreased the Simpson index and Shannon‐Wiener index of soil fungi. The possible reasons were speculated as follows: Firstly, compared to bacteria, fungi had larger individuals and were more susceptible to grazing disturbances. Secondly, according to disturbance theory, there was a nonlinear relationship between disturbance and resource and environmental heterogeneity. Moderate disturbance could increase species diversity, whereas excessive disturbance would decrease it. Under moderate grazing intensity, during the vigorous growth period of plants, a greater proportion of soil nutrients was allocated to plant growth and reproduction, resulting in fewer nutrients available for soil fungi. Under the HG treatment, both aboveground and belowground plant biomass were significantly reduced (Table 2), leading to a decrease in nutrients required for plant growth. However, the excretion process of livestock manure returned nutrients to the ecosystem, thereby providing more survival resources for soil fungi.

The impact of grazing on soil microbial community dynamics was a highly complex process that was closely related to plant types and also influenced by soil physicochemical properties and environmental factors (Wu et al. 2022). Variations in grassland types and soil texture contributed to the complexity of the relationship between soil microbial communities and soil biotic and abiotic characteristics under grazing. The results of this study indicated that soil pH and moisture were key factors affecting soil bacterial communities, while soil pH was a critical factor influencing soil fungal communities (Figure 5 and Table 3). This suggested that different factors mediated the effects of grazing on soil bacteria and fungi, which were consistent with some research findings under different grazing intensities (Cao et al. 2011; Wang et al. 2021). The possible reason was speculated to be that soil microorganisms were sensitive to changes in soil pH and water availability. Specifically, the alteration of soil acidity and alkalinity caused by the excretion of livestock manure and urine due to grazing led to changes in soil microbial community composition, potentially shifting from one dominant species to another (Levy‐Booth et al. 2016).

### 4.2 Impact of Different Grazing Intensities on Soil Microbial Community Functions

Microorganisms were not only an essential part of soil ecosystems but also possessed various functions, such as metabolism and nutrient cycling (Gao et al. 2021). In this study, a differential analysis of soil microbial functions was conducted. A total of 368 pathways were annotated using the KEGG database. A differential analysis of the top 10 metabolic pathways with the highest relative abundance revealed no significant differences among different grazing intensities. Jiao et al. (2022) conducted research on restored cropland ecosystems using metagenomic sequencing technology and found that core microbial communities played a crucial role in maintaining the functional stability of afforested ecosystems, with the “core functional characteristics” of microorganisms being relatively conserved. In this study, although soil microbial communities changed with different grazing intensities, their metabolic functions remained relatively conserved. This indicated that the soil microorganisms under different grazing intensities in this study could still maintain stable ecological functions when subjected to external environmental disturbances (Gao et al. 2021). Pang et al. (2021) demonstrated that the metabolic pathways of soil microorganisms were similar under different treatments, but there were slight differences in gene sequences within specific metabolic pathways. In this study, by comparing the relative abundances of different ko numbers in the nitrogen metabolism pathway, it was found that the relative abundances of some functional genes related to nitrogen metabolism (ko00910) were downregulated in the LG treatment, indicating that the LG treatment reduced soil nitrogen metabolism processes. This further reflected the research findings that the ecological functions of soil microbial communities were primarily metabolic functions (Hao et al. 2019). Specific functional genes of soil microorganisms regulate nitrogen‐cycling processes, and this characteristic also indirectly determines that microbial functional genes act as drivers for the conversion of various forms of nitrogen (Y. F. Wang et al. 2022). In this study, metagenomic‐sequencing technology was used to investigate the response of soil nitrogen metabolism functional genes to different grazing intensities. In this study, the soil nitrogen metabolism involved four pathways: dissimilatory nitrate reduction, assimilatory nitrate reduction, denitrification and nitrification. Among them, the denitrification pathway was detected with a higher frequency in the regional microorganisms, while nitrogen fixation (*nifH*) and anammox (*hzsA*) were not detected. This research outcome was consistent with the findings of Nelson et al. (2016), indicating similar characteristics of soil nitrogen metabolism pathways in the study area. The anammox process refers to the process in which related microorganisms use NO^2−^ as an electron acceptor to convert NH^4+^ into N_2_ through oxidation in an anaerobic environment, this process is considered an important pathway for nitrogen removal in natural environments and contributes to accelerating the global nitrogen‐cycling rate (Mulder et al. 1995). The anammox process mainly occurs in anaerobic environments, but the soil in the study area was unsuitable for the survival and development of such microorganisms (Humbert et al. 2010). Additionally, the nitrogen metabolism functional gene *narG* was detected under various grazing intensities. Nitrate reductase has many origins, such as periplasmic nitrate reductase and nitrite oxidoreductase. Among them, *narG* is often used as a marker gene for denitrifying microorganisms in the detected environment (John and Partha 2002). As an important structural gene of nitrogen‐fixing microorganisms, *nifH* was often used as a key indicator for detecting nitrogen‐fixing bacteria (Xu et al. 2014). This study did not detect biological nitrogen fixation pathways, possibly because the soil in the study area was inhospitable to the survival and reproduction of nitrogen‐fixing microorganisms. For instance, the total nitrogen content in the soil was relatively high, falling into the high‐nitrogen level. Excessive nitrogen content inhibits nitrogenase activity in nitrogen‐fixing microorganisms, as the nitrogen fixation process requires substantial energy, which in negatively impacts the functional genes associated with biological nitrogen fixation pathways (Zhang et al. 2013).

## 5 Conclusion

Grazing livestock exerted direct and indirect effects on soil microbial communities through foraging, trampling, and fecal and urinary excretion, thereby influencing the stability of alpine grassland ecosystems. The results of this study indicated that: (1) Grazing exerted a more significant impact on soil fungal communities than on soil bacterial communities. (2) Grazing increased the relative abundance of Cyanobacteria in the soil. (3) Soil pH and moisture content were the primary factors driving changes in soil bacterial and fungal communities. (4) Grazing altered the composition and structure of soil microbial communities but had a minimal impact on microbial functions. This study revealed the effects of different grazing intensities on the structure and function of soil microbial communities in alpine grasslands around Qinghai Lake on the Qinghai‐Tibet Plateau, providing a reliable theoretical foundation for the sustainable management of the region.

## Author Contributions

**Caicai Sun:** data curation (lead), formal analysis (lead), investigation (lead), writing – original draft (lead), writing – review and editing (lead). **Quanmin Dong:** supervision (lead), validation (lead). **Haitao An:** data curation (equal), investigation (equal), validation (equal). **Yuzhen Liu:** supervision (supporting). **Weidong Lv:** investigation (supporting). **Wenting Liu:** supervision (supporting). **Haiming Ji:** investigation (supporting). **Xiaoxia Yang:** project administration (equal), supervision (equal), validation (equal).

## Conflicts of Interest

The authors declare no conflicts of interest.

## Supporting information


Data S1.

## Data Availability

The raw data included in this study have been uploaded to Dryad and can be downloaded via the following link: http://datadryad.org/stash/share/wlQJ7dGcppHZO9W_YhX4BlZgH1SYnE9l‐San6JfoxSY.
