# Supplementary material for: Responses of Soil Microbial Communities and Functions in an Alpine Grassland of the Qinghai Lake Basin With Grazing Disturbance
Source: Ecol Evol. 2025 Mar 23;15(3):e71082. doi: 10.1002/ece3.71082 (PMC11930379; doi:10.1002/ece3.71082)
Supplement: Supplementary file 1 — Data S1. [file ECE3-15-e71082-s001.docx]

Table S1 Description of functional genes related to soil nitrogen metabolism

| KO | KEGG name | KO Description |
| --- | --- | --- |
| K15864 | nirS | nitrite reductase (NO-forming) / hydroxylamine reductase [EC:[1.7.2.1](https://www.genome.jp/entry/1.7.2.1) [1.7.99.1](https://www.genome.jp/entry/1.7.99.1)] |
| K00366 | nirA | ferredoxin-nitrite reductase [EC:[1.7.7.1](https://www.genome.jp/entry/1.7.7.1)] |
| K00372 | nasC, nasA | assimilatory nitrate reductase catalytic subunit [EC:1.7.99.-] |
| K00363 | nirD | nitrite reductase (NADH) small subunit [EC:[1.7.1.15](https://www.genome.jp/entry/1.7.1.15)] |
| K00362 | nirB | nitrite reductase (NADH) large subunit [EC:[1.7.1.15](https://www.genome.jp/entry/1.7.1.15)] |
| K00370 | narG, narZ, nxrA | nitrate reductase / nitrite oxidoreductase, alpha subunit [EC:1.7.5.1 1.7.99.-] |
| K00368 | nirK | nitrite reductase (NO-forming) [EC:[1.7.2.1](https://www.genome.jp/entry/1.7.2.1)] |
| K10945 | pmoB-amoB | methane/ammonia monooxygenase subunit B |
| K00367 | narB | ferredoxin-nitrate reductase [EC:[1.7.7.2](https://www.genome.jp/entry/1.7.7.2)] |
| K04561 | norB | nitric oxide reductase subunit B [EC:[1.7.2.5](https://www.genome.jp/entry/1.7.2.5)] |
| K02568 | napB | nitrate reductase (cytochrome), electron transfer subunit |
| K02567 | napA | nitrate reductase (cytochrome) [EC:[1.9.6.1](https://www.genome.jp/entry/1.9.6.1)] |
| K15876 | nrfH | cytochrome c nitrite reductase small subunit |
| K00360 | nasB | assimilatory nitrate reductase electron transfer subunit [EC:1.7.99.-] |
| K00374 | narI, narV | nitrate reductase gamma subunit [EC:[1.7.5.1](https://www.genome.jp/entry/1.7.5.1) 1.7.99.-] |
| K00371 | narH, narY, nxrB | nitrate reductase / nitrite oxidoreductase, beta subunit [EC:[1.7.5.1](https://www.genome.jp/entry/1.7.5.1) 1.7.99.-] |
| K03385 | nrfA | nitrite reductase (cytochrome c-552) [EC:[1.7.2.2](https://www.genome.jp/entry/1.7.2.2)] |
| K10944 | pmoA-amoA | methane/ammonia monooxygenase subunit A [EC:[1.14.18.3](https://www.genome.jp/entry/1.14.18.3) [1.14.99.39](https://www.genome.jp/entry/1.14.99.39)] |
| K10946 | pmoC-amoC | methane/ammonia monooxygenase subunit C |
| K02305 | norC | nitric oxide reductase subunit C |
| K00376 | nosZ | nitrous-oxide reductase [EC:[1.7.2.4](https://www.genome.jp/entry/1.7.2.4)] |

Table S2 Relative abundance of major genes in soil nitrogen metabolic pathways

|  | CK | LG | MG | HG |
| --- | --- | --- | --- | --- |
| **NirS** | **0.0031±0.00013a** | **0.0022±0.00047b** | **0.0029±0.00008ab** | **0.0032±0.00011a** |
| NirA | 0.0022±0.00007 | 0.0021±0.00006 | 0.0022±0.00005 | 0.0022±0.00006 |
| NasA | 0.0022±0.00008 | 0.0021±0.00005 | 0.0022±0.00005 | 0.0022±0.00006 |
| NirD | 0.0022±0.00007 | 0.0021±0.00006 | 0.0022±0.00005 | 0.0022±0.00006 |
| NirB | 0.0022±0.00008 | 0.0021±0.00006 | 0.0022±0.00005 | 0.0022±0.00006 |
| NarG | 0.0022±0.00008 | 0.0021±0.00006 | 0.0022±0.00005 | 0.0022±0.00007 |
| NirK | 0.0021±0.00008 | 0.0020±0.00008 | 0.0022±0.00006 | 0.0022±0.00006 |
| AmoB | 0.0021±0.00007 | 0.0020±0.00006 | 0.0021±0.00005 | 0.0022±0.00005 |
| NarB | 0.0013±0.00006a | 0.0011±0.00006b | 0.0013±0.00004 | 0.0013±0.00005a |
| NorB | 0.0021±0.00008 | 0.0020±0.00007 | 0.0021±0.00006 | 0.0021±0.00006 |
| NapB | 0.0020±0.00006 | 0.0019±0.00005 | 0.0020±0.00006 | 0.0021±0.00007 |
| NapA | 0.0020±0.00008 | 0.0018±0.00012 | 0.0020±0.00005 | 0.0021±0.00006 |
| NrfH | 0.0020±0.00008 | 0.0018±0.00010 | 0.0020±0.00006 | 0.0020±0.00006 |
| **NasB** | **0.0019±0.00009a** | **0.0016±0.00006b** | **0.0019±0.00005a** | **0.0020±0.00006a** |
| **NarI** | **0.0019±0.00009a** | **0.0016±0.00004b** | **0.0018±0.00005a** | **0.0019±0.00006a** |
| **NarH** | **0.0018±0.00008a** | **0.0015±0.00004b** | **0.0018±0.00006a** | **0.0019±0.00007a** |
| **NrfA** | **0.0018±0.00008a** | **0.0014±0.00004b** | **0.0017±0.00006a** | **0.0019±0.00006a** |
| **AmoA** | **0.0017±0.00009a** | **0.0013±0.00006b** | **0.0016±0.00007a** | **0.0018±0.00006a** |
| **AmoC** | **0.0017±0.00009a** | **0.0013±0.00006b** | **0.0016±0.00007a** | **0.0018±0.00006a** |
| **NorC** | **0.0015±0.00005a** | **0.0011±0.00004b** | **0.0015±0.00004a** | **0.0016±0.00006a** |
| **NosZ** | **0.0013±0.00006a** | **0.0011±0.00006b** | **0.0013±0.00004a** | **0.0013±0.00005a** |

Note: Bold values represent significant relationships. CK, No grazing; LG, Light grazing; MG, Moderate grazing; HG: Heavy grazing.

Fig. S1 Soil nitrogen metabolism pathway.
